# *Medicago Sativa* Defensin1 as a tumor sensitizer for improving chemotherapy: translation from anti-fungal agent to a potential anti-cancer agent

**DOI:** 10.3389/fonc.2023.1141755

**Published:** 2023-05-26

**Authors:** Raghu Pandurangi, Amol Karwa, Uma Shankar Sagaram, Katherine Henzler-Wildman, Dilip Shah

**Affiliations:** ^1^ Sci-Engi-Medco Solutions Inc (SEMCO), St Charles, MO, United States; ^2^ Mallinckrodt Pharmaceuticals, Hazelwood, MO, United States; ^3^ DeLuca Biochemistry Laboratories, University of Wisconsin, Madison, WI, United States; ^4^ Donald Danforth Plant Science Center, St Louis, MO, United States

**Keywords:** defensin, multi-drug resistance MDR, thioredoxin Trx, synergy, Doxorubicin

## Abstract

Plant defensins including *Medicago Sativa* defensin 1 (MsDef1) are cysteine-rich antifungal peptides which are known for potent broad-spectrum antifungal activity against bacterial or fungal pathogens of plants. The antimicrobial activities of these cationic defensins are attributed to their capacity to bind to cell membranes to create potentially structural defects tin the cell membranes to interact with intracellular target (s) and mediates cytotoxic effects. Our earlier work identified Glucosylceramide (GlcCer) of fungus *F. graminearum* as a potential target for biological activity. Multi-drug resistant (MDR) cancer cells overexpress GlcCer on the surface of plasma membrane. Hence, MsDef1 may have a potential to bind to GlcCer of MDR cancer cells to induce cell death. We have characterized the three-dimensional structure of MsDef1 and the solution dynamics using of ^15^N-labeled MsDef1 nuclear magnetic resonance (NMR) spectroscopy which showed that GlcCer binds MsDef1 at two specific sites on the peptide molecule. The ability of MsDef1 to permeate MDR cancer cells was demonstrated by measuring the release of apoptotic ceramide in drug resistant MCF-7R cells. It was also shown that MsDef1 activated dual cell death pathways ceramide and Apoptosis Stimulating Kinase ASK1 by disintegrating GlcCer and oxidizing tumor specific biomarker thioredoxin (Trx) respectively. As a result, MsDef1 sensitizes MDR cancer cells to evoke a better response from Doxorubicin, a front-line chemotherapy for triple negative breast cancer (TNBC) treatment. The combination of MsDef1 and Doxorubicin induced 5 to10-fold greater apoptosis *in vitro* MDR cells MDA-MB-231R compared to either MsDef1 or Doxorubicin alone. Confocal microscopy revealed that MsDef1 facilitates a) influx of Doxorubicin in MDR cancer cells, b) preferential uptake by MDR cells but not by normal fibroblasts and breast epithelial cells (MCF-10A). These results suggest that MsDef1 targets MDR cancer cells and may find utility as a neoadjuvant chemotherapy. Hence, the extension of antifungal properties of MsDef1 to cancer my result in addressing the MDR problems in cancer.

## Introduction

The development of multidrug resistance (MDR) is a serious clinical problem that is responsible for therapy failure, relapse of cancer and making tumors refractory to future treatments (1–3). Deactivation of cell death pathways (e.g., ceramide, apoptosis stimulating kinase, ASK1) (4) and avoidance of immune surveillance (5, 6) play major roles in desensitizing cancer cells to treatments irrespective of nature of treatments. As a result, high drug dose is needed to treat the cancer which in turn induces stemness into cancer cells, increases resistance, suppresses immune function, and enhances the off-target toxicity leading to side effects and treatment failure (7–10).

Despite its moderate to low efficacy and high off-target toxicity, chemotherapy is still the front-line treatment for most cancers (11, 12). The major problem with chemotherapy is that it kills only bulk non-stem cancer cells leaving behind resistant cells. This results in high disease recurrence [13% for kidney cancer, 36% for breast and almost 100% for brain cancer (13, 14)] and makes tumors refractory to future treatments (15). Dose related side effects such as acute lymphedema and low platelets may force discontinuation of treatment altogether creating more resistant cancer cells (16) and thus, causing a vicious circle. Sensitizing resistant tumor cells is known to evoke a better response from chemotherapy (17, 18). However, sensitizing tumor cells selectively using MDR biomarkers is of prime importance in order to make chemotherapy effective in lowering the side effects. This is true particularly for TNBC patients who have no option but chemotherapy with a >90% recurrence rate and a median survival of 13 months (19, 20). The situation is worse for African Americans with recurrence rates of >95%. Current targeted treatments for TNBC patients do not work since they lack biomarkers (e.g., ER, PR and HER2 negative) for which drugs are designed (21). That leaves non-specific anthracyclines (e.g., Doxorubicin, Paclitaxel) as sole options in spite of their high side effects and suppressed immune function (5, 6).

Cancer cells desensitize themselves to chemotherapy for their survival. For example, TNBC cells circumvent Doxorubicin effect by glycosylating lethal, apoptotic ceramide using glucosylceramide synthase (GCS) enzyme to non-apoptotic GlcCer which becomes a biomarker of MDR (22). In addition, GlcCer is consistently present at high levels in drug resistant tumors and in tumors taken from patients who are non-responsive to chemotherapy (23). GlcCer was found to be low in patients who responded to chemotherapy (24). Stemness of cancer cells increases as glycosylation increases (25). In fact, ceramide glycosylation selectively maintains the properties of cancer stem cells (CSCs) which is a serious clinical issue (25). Cancer cells also overexpress Trx and deactivate ASK1 cell death pathway (26–28) resulting in immune response suppression (29–31). Tumors with low Trx levels exhibit a better prognosis than tumors with high Trx levels (poorer prognosis, *P* < 0.001) for partial free survival (PFS) and for overall survival (OS) (32, 33). GlcCer and Trx are the two tumor specific biomarkers of resistance (34, 35). Hence, tumor sensitizers targeting GlcCer and Trx which act as immunoadjuvants are presently the unmet medical need in cancer therapy.

*Medicago sativa* Defensin1 (MsDef1) is a natural antifungal peptide (36) consisting of 45 amino acids and containing four disulfide bonds to form a folded protein. MsDef1 overexpressed in genetically modified potato wards off a Verticillium wilt disease caused by a fungal pathogen *Verticillium dahlia* (37). Ramamoorthy et al. (38) previously reported that a knockout of the GlcCer synthase gene, *gcs1*, in a fungal pathogen *Fusarium graminearum* blocked the antifungal activity of MsDef1 revealing the involvement of GlcCer in the mode of action (MOA) of this peptide. To date, no plant defensins have been shown to bind to MDR cancer cells and synergize with chemotherapies. None of the current MDR modulators (e.g., Eliglustat) target two tumor specific MDR targets.

Here, we report 3-dimensional structure of MsDef1 and determine its binding sites for its sphingolipid receptor GlcCer. We show that MsDef1 targets dual tumor specific targets namely MDR biomarker GlcCer in cancer cells and Trx, liberating ceramide and ASK1 protein from GlcCer and Trx respectively and in the process activating dual cell death pathways. We further show that MsDef1 synergizes with Doxorubicin and makes it effective at lower doses *in vitro*.

## Materials and methods

### Production of MsDef1

MsDef1 was produced using two methods. 1) It was produced recombinantly in *Pichia pastoris* as described previously (39). MsDef1 expressed recombinantly in *P. pastoris* was further purified by RP-HPLC using a reverse phase C18 column (Delta Pak Wat 011793, 15063.9 mm, 5 μM, 300 A) to obtain 95% purity and characterized by mass spectrometry. The purification yielded several species of MsDef1 which was separated by RT-HPLC (See **Figure S1**; **Supplementary Materials**). MsDef1 was dissolved in sterile double distilled water and its concentration was determined by using the BCA assay kit (Pierce, Rockford, IL). 2) Linear MsDef1 was chemically synthesized by using the standard peptide synthesizer (Apex 396 Parallel Synthesizer). Folding of the peptide was achieved through controlled air oxidation of the linear peptide. A solution of linear MsDef1 was dissolved in the double-distilled water in a test tube fitted with a probe through which air was bubbled through the solution for 36-48 hrs and monitored using mass spectrometry for a molecular ion corresponding to the oxidation of four S-S bonds. For example, 0.4 mg of linear peptide was dissolved in double distilled water in 50 mM phosphate buffer containing 1M Guanidinium-HCl at pH 7.5. The peptide sample was aliquoted at different time points: 0, 4h, 18h, 24h, 36 and 48h. Each peptide sample was desalted using C18 zip tip and run on LTQ-Orbitrap Velos by direct infusion. The samples were run with high resolution (60,000, LTQ-Velos Pro Orbitrap LC-MS/MS). The characterization includes purity by HPLC (> 96%), linear MsDef1 corresponding to deconvoluted mono isotopic mass 5191.23 and the fully folded MsDef1 corresponding to deconvoluted mono isotopic mass 5183.25. The exact difference 8 is due to the formation of four S-S bonds, a loss of 8 hydrogens. MsDef1 prepared by the slow oxidation method coincides with the one by recombinant method. The oxidation protocol was necessary for the production of large quantities of MsDef1 with correct molecular mass and folding for *in vivo* studies.

### Synthesis of 6-((N-(7-nitrobenz-2-oxa-1, 3-diazol-4-yl) amino) hexanoyl -MsDef1

The crude linear MsDef1 was dissolved in 20% methanol/8 M guanidinium hydrochloride and mixed with 6-((N-(7-nitrobenz-2-oxa-1, 3-diazol-4-yl) amino) hexanoyl (NBD) sphingosine in dark conditions. The mixture was stirred for 48 hrs before passing through the Sep-Pak and purified by RP-HPLC using eluents of H_2_O/0.1% TFA (eluent A) and acetonitrile/0.1% TFA (eluent B). The programmed elution profile 0–1 min, 100% A; 1–80 min, B is increased from 0–75% at a flow rate of 10 mL/min on preparative column (Spirit Peptide C18, 5 µm column, 19× 100 mm). Peptide purity was determined by an analytical HPLC monitoring peptide elution by absorbance at 220 nm. The conjugated peptide was folded using the air oxidation protocol described earlier. The mass of the conjugated peptide was determined to be 5745.9 (M+H) in agreement with the expected mass of the correctly folded NBD-MsDef1 conjugate.

### Structural analysis of ^15^N-labeled MsDef1 using NMR

^15^N-labeled MsDef1 was prepared as described previously (38). Briefly, *P. pastoris* cultures were grown overnight in buffered YNB media with no amino acids and 1.2% ^15^NH_4_Cl dissolved in 1M potassium phosphate (pH = 6.0), 500x biotin, 10% glycerol and then induced with 0.05% methanol every 24 h, according to the manufacturer’s directions (Thermo Fisher Scientific, CA, USA). The cultures were grown for 7 d at 29^0^ C, and cells were removed by centrifugation at 2,000g for 15 min. ^15^N-labeled MsDef1 was purified from the growth medium using CM-Sephadex C-25 cation-exchange chromatography and reverse-phase HPLC. The mass spec analysis of the labeled peptide revealed a single peak at 5254.03 (M+H) corresponding to the correctly folded ^15^N-labeled MsDef1.

NMR experiments were conducted on a Varian 700MHz spectrometer with HCN probe (backbone dynamics) and Bruker 600 MHz spectrometer with QCI cryoprobe (assignments and structure determination). Backbone (N and H_N_) and side chain protons were assigned for MsDef1 in aqueous buffer (25 mM HEPES, 20 mM NaCl, pH 6) at 25°C. The backbone amides were assigned using ^15^N-separated TOCSY and NOESY spectra with 60 ms and 150 ms mixing times, respectively. These experiments were acquired at 25°C with a 250 µM ^15^N-labeled MsDef1 in 90% H2O/10% D_2_O. Side chain protons were assigned using these spectra plus ^1^H-^1^H COSY, TOCSY, and NOESY spectra acquired for an otherwise identical unlabeled MsDef1 sample in 100% D_2_O. Backbone amide dynamics experiments were carried out using the same ^15^N-labeled peptide. R_1_, R_2_, and heteronuclear NOE experiments were performed using the standard pulse sequences. At least 8 timepoints were acquired for R_1_ and R_2_ measurements and heteronuclear NOEs were determined from an average of two independent experiments. Error estimates were based on the signal/noise ratio of each spectrum. Single exponential decays were fit to determine R_1_ and R_2_ rates using IgoPro (Wave metrics). Model free analysis was performed using fast MF and Model Free 4.15. Chemical shift changes upon interaction of MsDef1 with GlcCer were determined using the ^15^N-labeled peptide. Sixty micromolar d25-DPC (dodecyl phosphocholine, per deuterated acyl tail) was added to 0.25 µM MsDef1 in aqueous buffer to investigate the interaction of this peptide with DPC micelles. This sample was then added to lyophilized GlcCer, allowed to equilibrate for 2 h, and returned to the NMR tube to look for additional changes reflecting specific interaction with GlcCer.

### Isolation and purification of GlcCer from *F. graminearum*

Total lipids were extracted from 1g of *F. graminearum* mycelium and a glycolipid fraction containing GlcCer was purified with minor modifications as described previously (38). The mass spec analysis of the purified GlcCer showed a single peak (> 95% purity) at M+ ion 754.

### Cancer cell viability assays and determination of IC_50_

The MCF-7 cells were cultured in RPMI-1640 (Sigma, USA) while MDA-MB-231 was maintained in Dulbecco’s modified eagle medium (Sigma, USA). MCF10A cells (American Type Culture Collection, Manassas, VA) were cultured in DMEM/Ham’s F-12 (GIBCO-Invitrogen, Carlsbad, CA) supplemented with 100 ng/ml cholera toxin, 20 ng/ml epidermal growth factor (EGF), 0.01 mg/ml insulin, 500 ng/ml hydrocortisone, and 5% chelex-treated horse serum. The cells were cultured at 37 °C in a 90% humidified incubator with 5% CO_2_. When the cells were 80% confluent, they were sub-cultured to a fresh media. The SKOV3 cell line was obtained from the American Type Culture Collection (Manassas, VA) and cultured in DMEM growth media supplemented with 10% Fetal Bovine Serum, 2 mM Glutamine, and Pen-Strep antibiotic mixture. The flask containing cells were placed in an incubator which was maintained at a temperature of 37°C and 5% concentration of carbon dioxide (CO_2_) gas. Cardiomyocytes were derived from this engineered stem cell clone line as follows. Stem cell aggregates were formed from single cells and cultured in suspension in medium containing zebrafish bFGF (basic fibroblast growth factor) and fetal bovine serum. Upon observation of beating cardiac aggregates, cultures were subjected to blasticidin selection at 25 ug/ml to enrich the cardiomyocyte population. Cardiomyocyte aggregate cultures were maintained in Dulbecco’s modified Eagle’s medium (DMEM) containing 10% fetal bovine serum during cardiomyocyte selection through the duration of the culture prior to cryopreservation. At 30 to 32 days of culture the enriched, stem cell-derived cardiomyocytes were subjected to enzymatic.

dissociation using 0.5% trypsin to obtain single cell suspensions of purified cardiomyocytes, which were >98% cardiac troponin-T (cTNT) positive. These cells (iCell1Cardiomyocytes) were cryopreserved and stored in liquid nitrogen before delivery to Ionic Transport Assays from Cellular Dynamics International, Madison, WI.

Doxorubicin resistant MCF-7R and MDA-MB-231R cells were grown in DMEM 10% FBS with increasing concentrations of Doxorubicin as described by Bielawski et al. (40). Cells were seeded and exposed to increasing concentrations of Doxorubicin (10 nM to 100 nM). MCF-7 or MDA-MB-231 cells were seeded in cell culture flasks and after 24 h, cells were trypsinized, counted viable cells and reseeded into a new culture flask before adding Doxorubicin again. Cells were considered chemoresistant when at a particular concentration did not cause cell death. Generally, it took 6-7 passages to get Doxorubicin resistant cells.

Cell viability was evaluated by the standard MTT (3,4,5-dimethylthiazol-2-yl)-2,5-diphenyltetrazolium bromide, Sigma) method. All cancer cells (MDA-MB-231, MCF-7, MCF-R, Hela, MCF-10A epithelial breast cells, induced pluripotent stem cells derived cardiomyocytes (iPSc) were added to the wells of 96-well flat-bottom plates at the density of 2×10^4^ per well, allowed to attach overnight, and treated with different concentrations of MsDef1 (1-100 µM). After 24 h, 10 µl of MTT solution was added to each well and the plate was incubated at 37°C for 3 h. After removing the media, 200 µl of isopropanol were added to dissolve the crystals. Absorbance was read at 550 nm in an ELISA plate reader (Sunrise, Tecan, Milan, Italy), and the results are expressed as relative change with respect to the controls set as 100%. For cardiomyocytes, human iPSCs were used (Ionic Transports, St Louis).

### Thioredoxin assays using western blots

The Trx Western blotting was performed as described previously, with minor modifications (39). Briefly, 3 x 10^6^ cells were lysed in G-lysis buffer (50 mM Tris HCl, pH 8.3, 3 mM EDTA, 6 M guanidine–HCl, 0.5% Triton X-100) containing 50 mM iodoacetic acid (IAA; pH 8.3). For each experiment, control plates, for identifying Trx redox state bands in the Western blot, were also incubated with 2mM H_2_O_2_, for 10 min at room temperature, before incubation with 50 mM IAA. Subsequently, the lysates from all cells were incubated in the dark for 30 min with the IAA. The lysates were then centrifuged in G-25 micro spin columns (GE Healthcare). Protein was quantified from the eluent using the Bradford protein assay, as previously described (41–45).

### Ceramide assessment

Ceramide was extracted from cancer cells using the procedure described by Bielawski et al. (40). Briefly, drug resistant MCF-7R cells were grown on 10 cm plates containing 2–5 X10^6^ cells per plate, washed with 10 mL of phosphate buffer saline (PBS). The cells were scraped with 1 mL of methanol and transferred into a 5.5 mL glass vial with either aluminum or Teflon-sealed caps (SKS Science). Cells were sonicated for 60 min in a bath sonicator, and 100 µL of the solution was taken for protein concentration measurements. 50 µL of a 50 ng/mL C17 standard solution was added to the remaining cells in the glass vial to obtain a final concentration of 10 ng/mL of C17 as the internal standard (IS). At the end of the extraction procedure, 2 mL of chloroform was added to the cell suspension. After vortexing for 5s followed by 30 min of sonication, the cell lysates were spun for 5 min at 3000 rpm. Then, the lower layer containing chloroform was transferred to a new tube using a 1 mL glass pipette, leaving the upper layer (containing methanol) and the middle layers (containing proteins) (~75% extraction efficiency for this first extraction) and repeated twice to get > 92% extraction. The extracted solution was dried under nitrogen gas, reconstituted in acetonitrile before subjected to HPLC analysis (Agilent 1200, Agilent Technologies, Santa Clara, CA, RP column, 50 X 2.0 mm, 3 mm), Mobile phase A consisted of 0.1% formic acid and 25 mM ammonium acetate in water and mobile phase B consisted of 100% acetonitrile.

### Permeability assays

Resistant TNBC cancer cells MDA-MB-231R and ovarian SKOV3 cells were incubated with 20 µg/ml, 6.6 µg/mL, or 2.2 µg/ml of NBD-MsDef1 for 15 min at 37° C in a total volume of 200 µl. The excess NBD-MsDef1 was washed off with 1xPBS and cells were fixed with 4% paraformaldehyde to be imaged using the Nikon Confocal Microscope (TE 2000-E, PES). In order to determine the specificity of NBD-MsDef1 internalization, labeled cells were washed with water 3 times before imaging them. For assessing Doxorubicin influx in resistant MDA-MB-231 TNBC cells, autofluorescence of Doxorubicin was measured using confocal microscopy at emission wavelength of 595 nm post excitation with a 470 nm laser. For enhanced uptake of Doxorubicin by MDA-MB-231R cells post MsDef1 treatment, about 35,000 cells/well were plated in 8-well chambered lab-tek 2 slides and allowed to grow overnight. Cells were treated with 20 µ M of MsDef1, 3 µM Doxorubicin and a combination of 20 µ M MsDef1 and 3 µM Doxorubicin in separate batches for 4 h before examined by confocal microscopy (Nikon A1i laser scanning confocal microscope). Images at 1-min intervals were collected and analyzed. All fluorescence images were analyzed, and the background subtracted with ImageJ software. Pearson’s coefficient was quantified using the Colocalisation Analysis plugin for ImageJ.

### MsDef1 stability

The Simulated Gastric Fluid (SGF) assay was performed as described by Fu et al., 2002 with some modification. Tests were performed in 500 μL of SGF (200 mg NaCl, 0.2% pepsin, pH 2) in glass tubes in a 37°C water bath with continuous stirring of the enzyme reaction. After 2 min preincubation, the assay was started by the addition of 25 μL of MsDef1 peptide (5 mg/ml) to each vial containing SGF, SGF without pepsin or ultrapure water. Five mg/ml bovine serum albumin (minimum 98%, A-7030, Sigma Aldrich) in ultrapure water was used as the positive control for pepsin digestion. Protein samples were analyzed for degradation by SDS PAGE followed by staining for 1 h and extensive washing with ultrapure water.

### Synergy studies

The percentage of apoptotic cells was determined by using TUNEL assay according to the manufacturer protocol. Briefly, cells were fixed with 1% paraformaldehyde at 4^0^C, followed by two washing with PBS. Cold 70% ethanol was added to the cell pellet. Cells were then incubated at −20°C for permeabilization. After washing with PBS, cells were incubated with staining solution containing TdT enzyme and fluorescein-dUTP for 60 min at 37°C. Samples were washed with rinse buffer and resuspended in 500 ml of propidium iodise/RNase solution for flow cytometry analysis.

## Results

MsDef1 is a 45-amino acid cysteine-rich peptide predicted to form four disulfide bonds was identified for its antifungal activity against filamentous plant pathogens was first reported in 2000 by Gao et al (46). Some defensins bind to specific sphingolipids with high affinity that are localized in the fungal cell wall and plasma membrane of their target fungi (47–49). Sphingolipids serve as second messengers for regulating cell growth, cell survival and death (50). Mechanistic studies suggest that MsDef1 binds to GlcCer in the cell wall of the fungal pathogen *F. graminearum*. A Gcs1 knock-out mutant (ΔFg*gcs1)* lacking GlcCer synthase activity and a depleted in GlcCer displays strong resistance to MsDef1. Understanding the binding of the specific amino acid residues of MsDef1 with MDR biomarker GlcCer opens an opportunity to design small peptide based drugs for a potential to treat MDR cancer. Here, we have determined the structural basis for the engagement of GlcCer by ^15^N labeled MsDef1 using ^15^N longitudinal relaxation (T1) and ^15^N-^1^H Nuclear Overhauser Effect (NOE) in solution dynamics NMR.

### Three-dimensional structure of MsDef1

**Figure 1:**
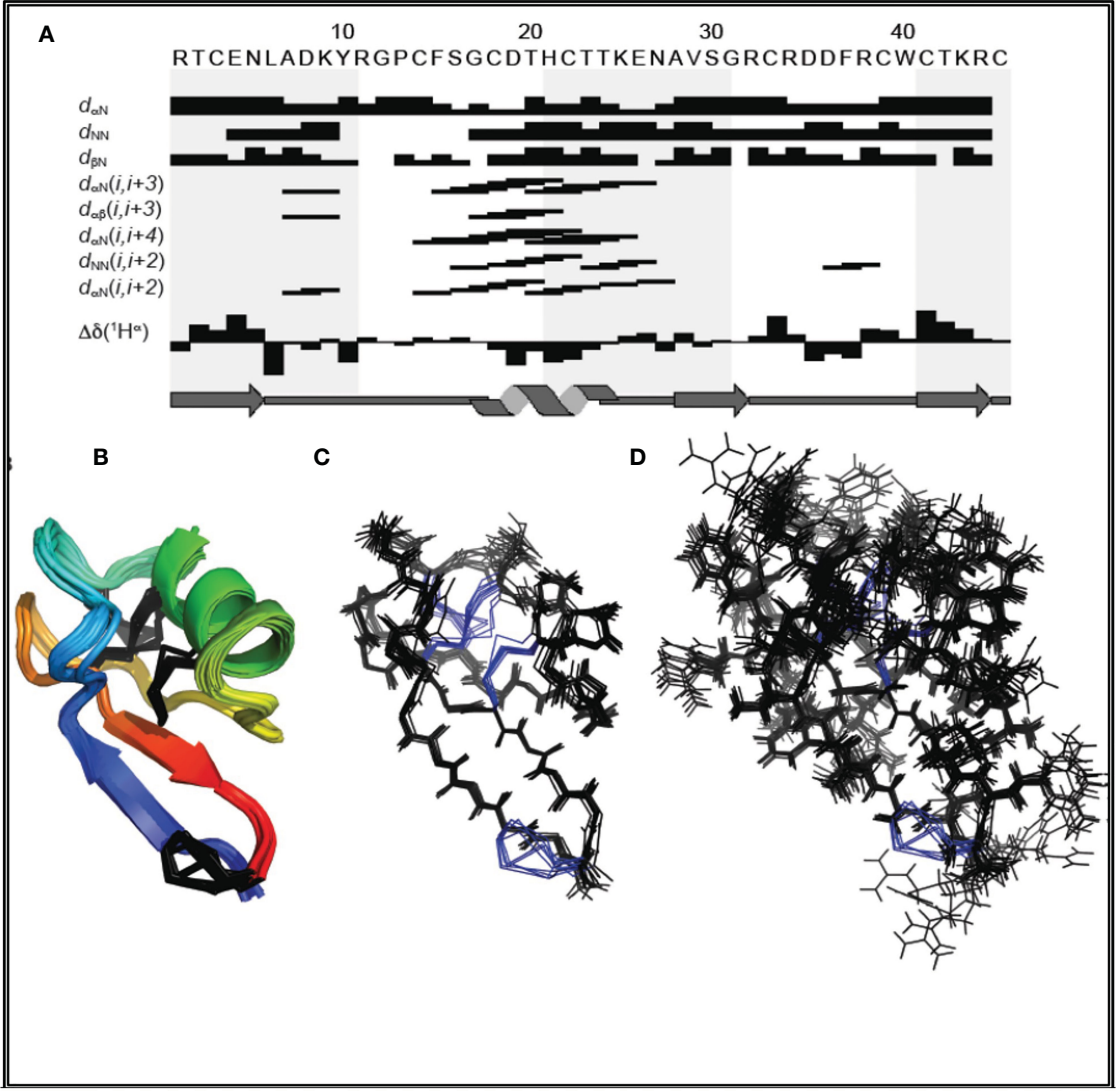
**(A)** Amino acid sequence of MsDef1 and survey of NMR data used to identify secondary structures. The sequential and medium range weak, medium, and strong NOEs are represented by the heights of the bars. The positions of β1, α-helix, β2 and β3 are shown relative to the amino acid sequence of MsDef1. **(B)** Backbone superposition of the 20 energy-minimized structures of MsDef1 showing the global fold and secondary structures. The disulfide bonds are shown in black. **(C)** Stereo view of the backbone atoms (N, C and O) of the structures in **(B)**. **(D)** Backbone superposition of the ensemble of structures showing the amino acid side chains.

The parameters characterizing the structure of MsDef1 are shown in **Table 1**. The structure of this peptide was derived from 981 distance constraints derived from 216 sequential, 61 short range, 38 medium range, 204 long range NOEs and 12 hydrogen bonds. **Figure 1** is a superposition of the final ensemble of structures calculated for MsDef1 with a single cartoon representation of this ensemble. This ensemble of structures results from 20 lowest energy structures from 80 calculated structures. The mean rmsd is 0.99 Å for backbone heavy atoms and 0.52 Å for all heavy atoms, respectively. Not surprisingly, MsDef1 has a highly compact structure which consists of one α-helix (α1=Cys18-Thr23) and three anti-parallel β-strands (β1=Thr2-Leu6, β2=Val29-Arg32, β3=Cys39-Arg44). The structure is stabilized by the presence of four disulfide bonds with Cys3-Cys45, Cys14-Cys33, Cys18-Cys39, Cys22-Cys41 configuration. The core structure is almost identical to the homology-based structure of MsDef1 published earlier (51) and is similar to other plant defensins with regard to folding and locations of the four disulfide bonds (52). Statistics from structural calculations are tabulated in **Table 1**.

**Table 1:** Statistics from Structure Calculation.

Parameter	Value
Number of Restraints	
NOE (total)	981
Sequential	216
Short-range	61
Medium-range	38
Long-range	204
Dihedral angles^a^	67
H-bonds	12
Energy (kcal/mol)	
Total	-1100.8 ± 85.5
Bond lengths	2.0 ± 0.1
Bond angles	23.6 ± 0.7
Impropers	4.2 ± 0.3
Van der Waals	-260.3 ± 51.6
Dihedral angles	207.7 ± 1.6
Electrostatics	-1077.9 ± 62.2
NOEs	187.1 ± 140.0
No. of NOE violations >0.5 Å (Å)	3 ± 2.3
No. of dihedral angle violations >5° (°)	0
RMS Deviations	
NOEs (Å)	0.059 ± 0.024
Dihedral angle restraints (°)	2.11 ± 0.05
Ideal bond lengths (Å)	0.0017 ± 0.0001
Ideal bond angles (°)	0.355 ± 0.005
Ideal improper angles (°)	0.270 ± 0.011
Backbone atoms (Å)	0.26 ± 0.04
	0.15 ± 0.02^b^
Heavy atoms (Å)	0.99 ± 0.09
	0.52 ± 0.09^b^
All atoms (Å)	
Ramachandran Plot (%)	
Most favored regions	76.2
Additional allowed regions	20.9
Generously allowed regions	2.9
Disallowed regions	0.0

Results from ensemble of 20 lowest energy structures from 80 calculated structures.

a ϕ and ψ torsion angles restraints derived from ^3^J_HNHα_ couplings directly measured from COSY spectra and chemical shift index analyzed with TALOS+.

b values for secondary structure elements only.

### MsDef1 binds to GlcCer “in situ”

**Figure 2:**
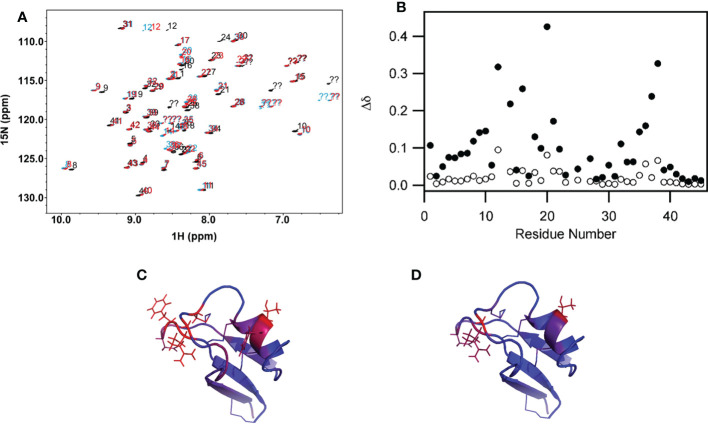
**(A)** Backbone region of the ^1^H-^15^N HSQC spectrum showing chemical shift changes in MsDef1 (black spectrum, aqueous buffer) upon addition of the DPC micelles (blue spectrum) and further addition of GlcCer (red spectrum). Significant peak shifts upon addition of the lipid-like detergent DPC indicates that MsDef1 does interact with the micelles. Addition of GlcCer causes additional chemical shift changes in a smaller subset of residues indicating that GlcCer interacts with MsDef1 in a more localized region. **(B)** Quantification of the chemical shift changes in MsDef1 upon addition of lipid. $\Delta \text{d}={\left(\Delta {\text{d}}_{\text{H}}^{\;2}+0.1*\Delta {\text{d}}_{\text{N}}^{\;\;3}\right)}^{1/2}$ . Significant chemical shift changes are observed in two regions upon addition of micelles of the lipid-like detergent, DPC (solid symbols, DPC versus aqueous buffer). Smaller additional changes are observed in a few localized sites upon further addition of GlcCer (open symbols, DPC + ceramide versus DPC only). **(C)** Chemical shift mapping of MsDef1-GlcCer interactions. **(A)** Residues with chemical shift changes upon association with DPC micelles. The color scale extends from blue (no change, Δd=0) to red (significant chemical shift change, Δd≥0.25). **(D)** Residues with additional chemical shift changes upon addition of GlcCer to the DPC micelles. The same color scale is used but with a narrower range (0≤Δd ≤ 0.10). One face of the molecule has significant backbone chemical shift changes upon addition of micelles indicating that this face interacts with DPC. GlcCer interacts with a smaller, more localized subset of the residues that interact with the lipid-like detergent micelles.

The solution dynamics of MsDef1 with GlcCer was assessed using chemical shift perturbation, ^15^N longitudinal relaxation (T1) and ^15^N-^1^H NOE. Upon binding of ^15^N–labeled MsDef1 with GlcCer with d25-DPC micelles, significant peak shifts occurred and upon addition of GlcCer additional chemical shift changes in a smaller subset of residues occurred (**Figures 2A, B**). Our results revealed that MsDef1 binds to GlcCer at two regions: amino acids between residues 12-20 and residues 33-40 (red color, **Figures 2C, D**). This is consistent with our earlier finding that mutation of Arg38 to Gln38 resulted in a complete loss of the antifungal activity of MsDef1 against *F. graminearum* (39). Binding of MsDef1 to GlcCer is the first premise on which MsDef1 is proposed as a targeted tumor sensitizer.

### MsDef1 regenerates ceramide from GlcCer

**Figure 3:**
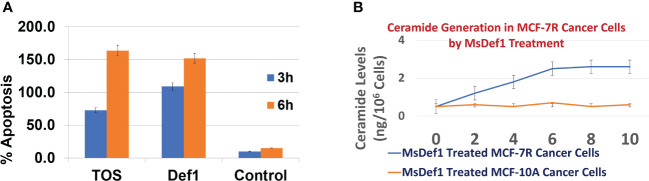
**(A)** Extent of apoptosis in GlucCer positive MDR MCF-7R cells treated with Def1 (20 μM) compared to positive control α-Tocopheryl succinate (TOS, 20 μM) and GlucCer negative normal MCF-10A breast epithelial cells, **(B)** Ceremide regeneration from GlucCer positive MCF-7R cells treated with Def1 (20 μM) compared to GlucCer negative normal MCF-10A breast epithelial cells.

Revamping ceramide pathway is important for those drugs which mediate the cell death through ceramide pathway (e.g., Doxorubicin). Changes in the liberation of ceramide in Doxorubicin resistant MCF-7R cells were measured at Lipidomics Shared Resource, Medical University of South Carolina (MUSC), using high performance liquid chromatography-mass spectrometry (LC-MS/MS) as previously described by Jacek et al. (40). Preliminary studies (**Figure 3B**) showed an enhanced accumulation of ceramide until 6 hrs of treatment with 20 μM MsDef1 in GlucCer positive MCF-7R (resistant) breast cancer cells compared to normal breast epithelial GlucCer negative control cells (MCF-10A). Ceramide is known for inducing apoptosis in cancer cells exemplified by Doxorubicin which goes through ceramide pathway mechanistically. Hence, apoptosis induced by ceramide released was also measured in MCF-7R cells which showed an order of magnitude higher than for normal cells (MCF-10A) which are GlucCer negative at 3 and 6 hrs of treatment (**Figure 3A**). The enhanced accumulation of ceramide in response to MsDef1 treatment was similar to that observed upon treatment with 20 μM α-tocopheryl succinate (TOS) (53) serving as a positive control. Reactivation of ceramide pathway is important since it is an effective sensitizing strategy in overcoming the resistance in metastatic colon and breast cancers *in vivo* (54). The enhanced accumulation of ceramide upon MsDef1 treatment in cancer cells selectively compared to normal breast cells is the second premise on which MsDef1 is proposed as a MDR targeted tumor sensitizer.

### MsDef1 oxidizes tumor specific biomarker Trx

**Figure 4:**
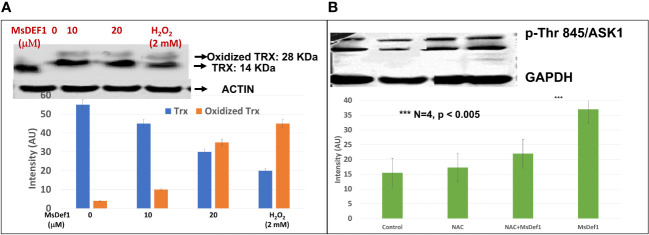
**(A)** MsDef1 oxidizes Trx at 20 μM dose compared to positive control H_2_O_2_ (2 mM) & presumably activates ASK1 cell death pathway in MDA-MB-231-R TNBC cells, N=4, p < 0.05, **(B)** Immunoblot analysis of phosphorylation of Threonine-845 residue of ASK1 Protein in response to the treatment of Def1 in MDA-MB-231 cells: 1. Control, 2. N-Acetyl-Cysteine (NAC, 5 mM)/60 mins, 3. NAC (5 mM) + Def1 (50 μM) for 60 min, 4. Def-1 (50 μM) alone for 60 minutes, N=4, P < 0.005, GAPDH: Internal Control. *** Statistically siginificant.

Several disulfide-linked peptide derivatives are shown to oxidize Trx, a tumor specific biomarker (54–60). Trx is known to be involved in development of Doxorubicin resistant cells by deactivating ASK1-pathway. MsDef1 was anticipated to interact with Trx due to its four disulfide bonds and availability of SH groups on Trx protein. Hence, further studies were conducted in cancer cells (e.g., TNBC, MDA-MB-231) using MsDef1. Doxorubicin resistant MDA-MB-231-R cells were treated with 0, 10 and 20 μM MsDef1 or 2 mM H_2_O_2_ (positive control). **Figure 4A** clearly indicates the oxidation of Trx at 20 μM MsDef1 by showing Trx peptide band at 28 KDa compared to untreated control which showed Trx band at 14 KDa. The oxidation of Trx by MsDef1 is similar to that observed with positive control 2 mM H_2_O_2_ (N=4, p < 0.05). This is the third premise on which MsDef1 is proposed as a targeted tumor sensitizer.

### MsDef1 disrupts Trx-ASK1 complex to activate ASK1 cell death pathway and induces apoptosis in resistant cancer cells

The oxidation of Trx is known to release ASK1 from the Trx complex through phosphorylation of several amino acid residues (33). We corroborated Trx oxidation data (**Figure 4A**) by assessing the phosphorylation status of Thr845 residue of ASK1 protein using specific antibody phosphorylation kit **(**Santa Cruz Biotech, CA). Fas resistant triple negative MDA-MB-231R cancer cells were treated with a) 50 μM MsDef1 for 60 minutes, b) 5 mM N-acetyl cysteine (NAC) as a negative control and c) NAC treated cells plus 50 μM MsDef1. **Figure 4B** showed a significant increase in phosphorylation of Thr845 residue (lane 4) upon treatment with MsDef1 compared to the solvent control (lane 1) and the NAC control (lane 2, N= 4, p <0.005). NAC is an FDA approved drug which inhibits phosphorylation of Thr485 of ASK1 protecting cells from death. MsDef1 induces significantly higher phosphorylation of Thr485 of ASK1 (lane 4) than it does even in presence of NAC (lane 3). These results indicate that MsDef1 might have targeted tumor specific Trx and activates ASK1 cell death pathway. Trx inhibitors with disulfide bonds are known to reactivate ASK1 pathway and sensitize cancer cells to chemotherapy similar to MsDef1 (55–60).

### MsDef1 permeates resistant cancer cells

**Figure 5:**
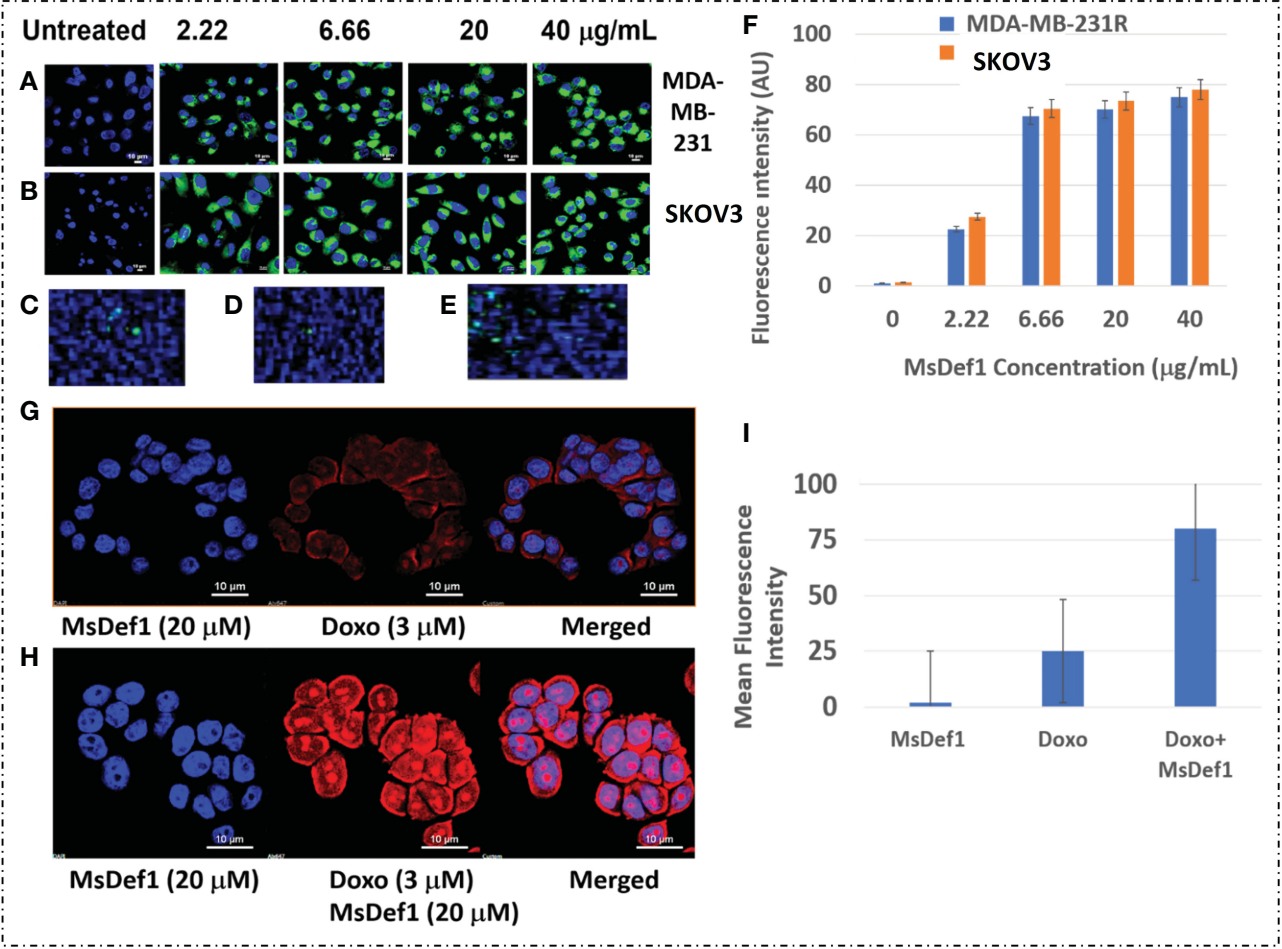
**(A)** NBD-Def1 permeates resistant **(A)** TNBC MDA-MB-231R Cells **(B)** SKOV3 Cells while, uptake is low for **(C)** Normal Epithelial Breast Cells **(D)** Fibroblasts & **(E)** Scrambled Def1 indicating selectivity, **(F)** Graphic representation of MsDef1Def1 uptake (N= 4, p < 001), **(G)** Effect of MsDef1Def1 on doxorubicin influx into MDR cancer cells MDA-MB-231R by confocal microscopy, N = 4, P < 0.002. Cells were stained with Hoechst 3325 and doxorubicin fluorescence was visualized at 488 nm, **(H)** Quantitative intrinsic mean fluorescence intensity of Doxorubicin. **(I)** Quantification of Uptake of Doxorubicin through Mean Fluorescence Intensity (AU).

Defensins are cationic peptides known for creating irreversible structural defects on the cell membranes with pore formation similar to CPPs (61). However, the specificity of MsDef1 to MDR tumor cells needs to be tested. Optical marker 6-((N-(7-nitrobenz-2-oxa-1, 3-diazol-4-yl) amino) hexanoyl (NBD) sphingosine was modified with linear MsDef1 before it was cyclized through oxidative protocol developed by us. The modified NBD-MsDef1 showed IC_50_ (12 μM) similar to the native MsDef1 indicating that the modification of the peptide did not alter its biological activity by a large margin. Confocal microscopy studies on MsDef1-NBD incubated with resistant TNBC MDA-MB-231R and ovarian SKOV3 cells respectively showed a significant uptake of MsDef1-NBD (**Figures 5A, B**) compared to untreated tumor cells while, normal epithelial breast cells (MCF-10A) and fibroblasts cells (**Figures 5C, D**) did not take up MsDef1 even at 5-fold higher dose of MsDef1 at 200 mg/mL. Simple washing of stained cells did not reduce the fluorescence intensity originated from NBD which confirms trapping of MsDef1 inside the cancer cells. Permeation of membrane compromised fungal cells by MsDef1 was recently reported by us using optical marker DyLight 550-MsDef1 (39). The low uptake of the scrambled MsDef1-NBD by tumor cells (**Figure 5E**) established the potential selectivity of MsDef1 action for tumor cells. **Figure 5F** shows accumulation of NBD-MsDef1 with respect to concentration in cancer cells graphically.

### Uptake of MsDef1 in resistant TNBC cells

MsDef1 presumably compromises MDR cell membrane integrity by disintegrating GlucCer to ceramide and allowing internalized MsDef1 to interact with the intracellular Trx. That is expected to allow a better perfusion of drugs influx into the cancer cells. The hypothesis was corroborated by measuring the influx of Doxorubicin by MsDef1 in MDR cancer cells through the intrinsic fluorescence of Doxorubicin at 488 nm using confocal microscopy. **Figures 5G, H** demonstrates a significant increase in the fluorescence intensity of Doxorubicin in MDA-MB-231R cells after treatment with 20 μM MsDef1 for 6-12 hr. compared to Doxorubicin alone. The increase in the fluorescence intensity showed uptake of Doxorubicin 3-fold more in presence of MsDef1 than in its absence **Figure 5i**. This result confirms the poor tumor penetration limitation of Doxorubicin on its own and may explain a potential pore formation of cancer cell membranes and the synergistic effect of MsDef1 allowing Doxorubicin to perfuse better into the tumor cells.

### MsDef1 exhibits antitumorigenic activity

**Figure 6:**
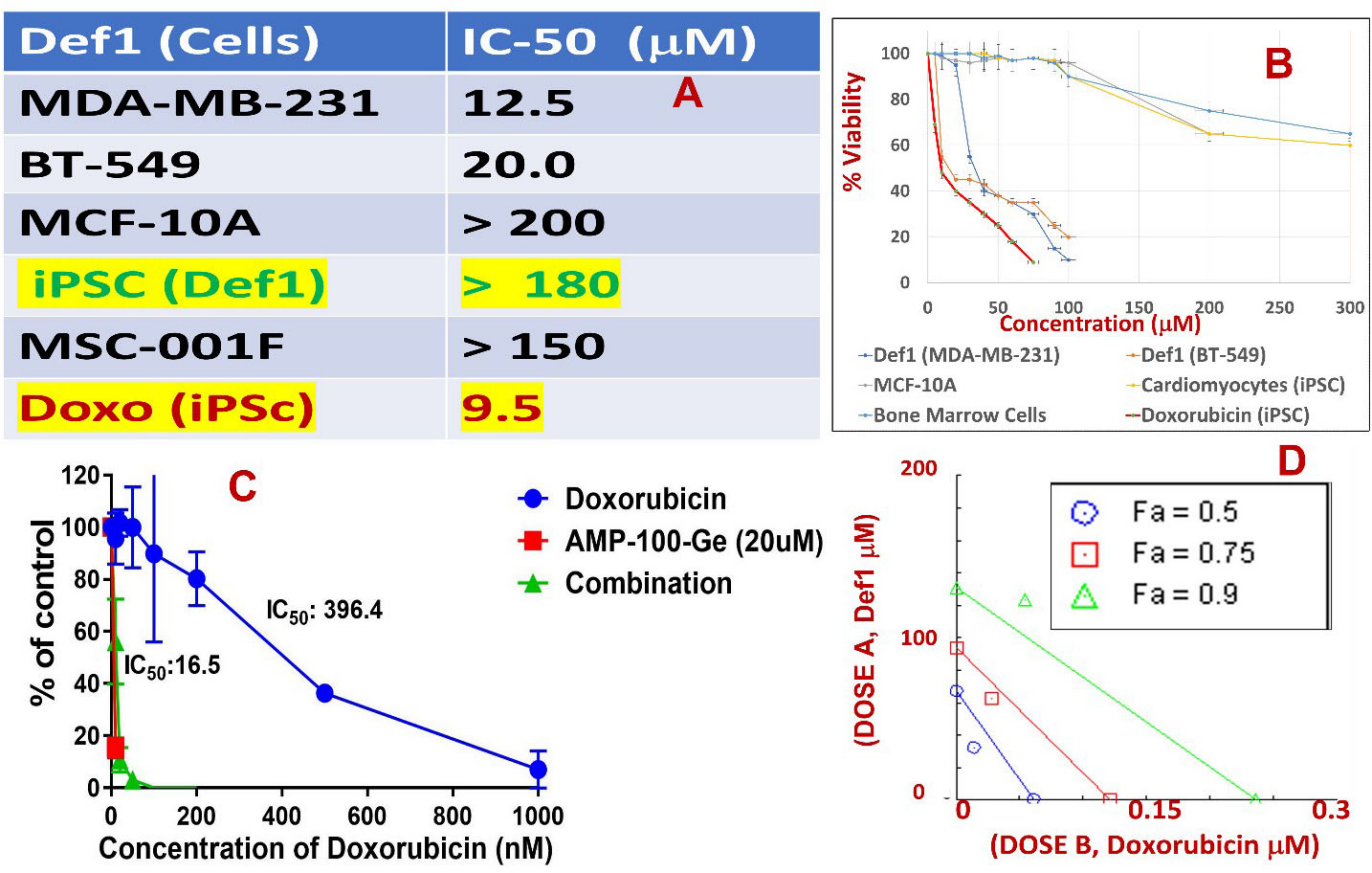
**(A)** Tabulated IC_50_ values of MsDef1 in MDA-MB-231, BT-549 cancer cells compared to normal cells MCF-10A, induced pluripotent stem cell derived cardiomyocytes (iPSCs), bone marrow cells (MSC-001F) and Doxorubicin. **(B)** The IC_50_ values were derived from viability assays. N=3, p < 0.05, Note: IC-50 values for normal cells by Def1 is ~ 10-15 times lower compared to cancer cells indicating better safety profile for Def1 *in vitro*, **(C)** IC_50_ curve for a) Def1 and b) combination of Def1 at 20 μM as a function of Doxorubicin dose in MDA-MB-231 cells, N= 4, p< 0.002. **(D)** Combination Index calculation for Def1 and doxorubicin in MDA-MB-231 cells: fa = fractional killing of cells. CI < 1.00 Synergistic, CI = 1.00 Additive, CI > 1.00, Antagonist.

Table in **Figure 6A** shows IC_50_ values for MsDef1 in several GlcCer and Trx positive cancer cells. These values are in the similar range as those of many chemotherapeutics (62–65). Several similar defensin type of molecules derived from natural sources also showed IC_50_ values in similar range (66, 67). However, none of them has been shown to have multiple characteristics of MsDef1 including liberation of ceramide, oxidation of Trx and synergy with chemotherapeutics. It should be noted that MsDef1 targets cancer cells *in vitro* (e.g., MDA-MB-231, MDA-MB-231R, HeLa, BT-459) while sparing normal epithelial breast cells (e.g., MCF-10A), bone marrow cells (MSC-001F) and cardiomyocytes (iPSC), an attribute important in determining the potential side effects of MsDef1 if it moves to the clinical phase. It should be noted that Doxorubicin, the first line treatment for TNBC, has IC_50_ value of 9.6 μM in iPSC cardiomyocytes compared to >180 μM for MsDef1 indicating a better safety profile for MsDef1. **Figure 6A** shows the tabulation of IC_50_ values for varieties of cancer cells which are GlucCer positive (e.g., MDA-MB-231, MDA-MB-231R, HeLa, BT-459) and normal cells including bone marrow cells (MSC-001F) and iPSc cardiomyocytes which are in general get severely affected by chemotherapy. It is to be noted that Doxorubicin kills normal cells at relatively lower doses (~ 9.6 μM), while MsDef1 is relatively safer even at > 200 μM (**Figure 6B**). On the contrary, MsDef1 kills cancer cells at lower μM similar to Doxorubicin potency. The combination of MsDef1 and Doxorubicin reduced IC_50_ values significantly from 396.6 nM for Doxorubicin to 16.5 nM indicating the synergy between MsDef1 and Doxorubicin (**Figure 6C**). The data is further confirmed by the measurement of combination index which was <1.00, hallmark of synergy (**Figure 6D**).

### MsDef1 synergizes with Doxorubicin to enhance apoptosis in cancer cells

**Figure 7:**
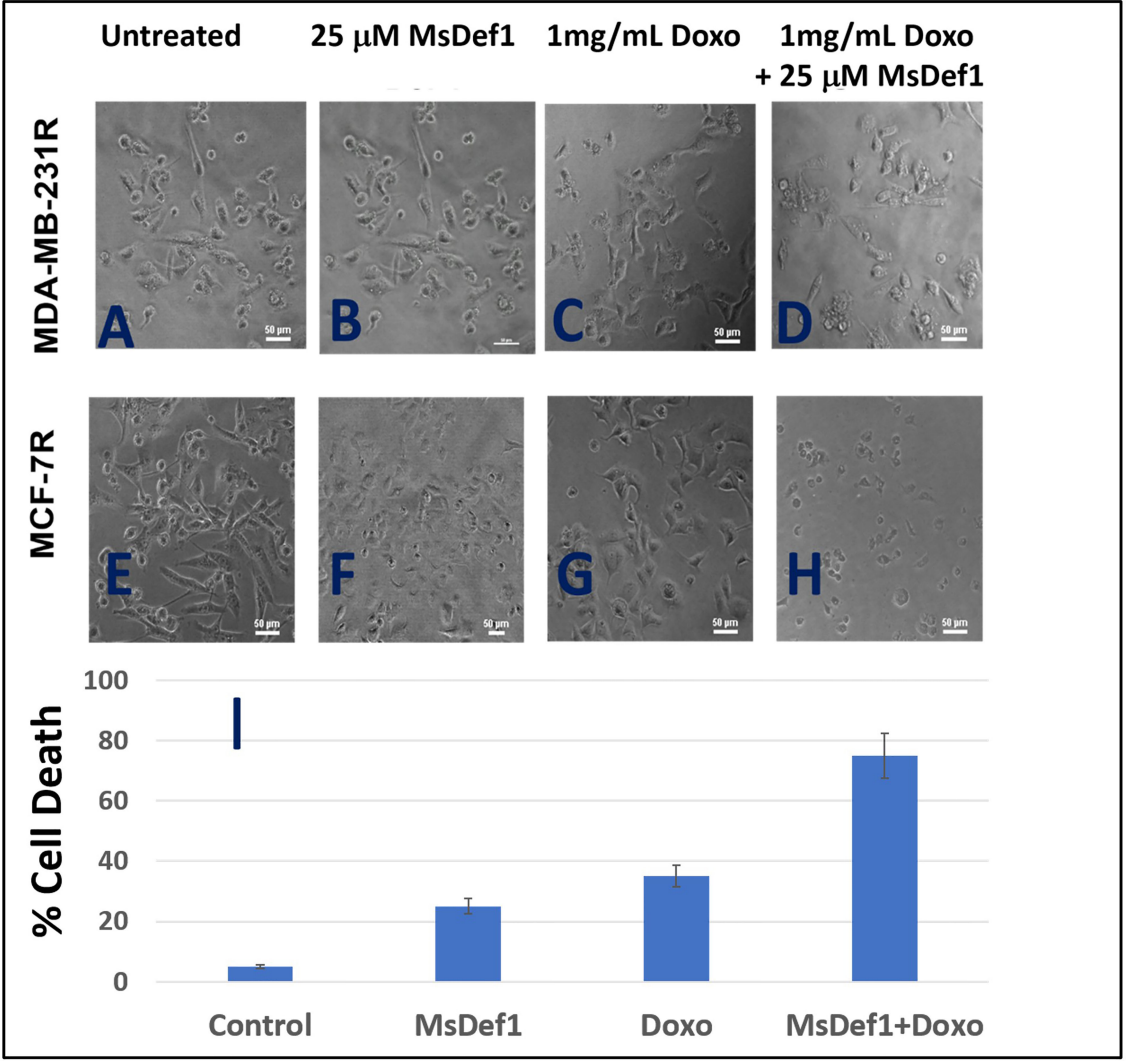
MsDef1 synergizes with doxorubicin in resistant MDA-MB-231R and MCF-7R cells **(A–D)** Micro photographs of **(A, E)** control, **(B, F)** Def1 alone, **(C, G)** Doxorubicin alone and **(D, H)** Combination followed by **(I)** quantification of apoptosis, N = 4, p < 0.007.

Although MsDef1 is cytotoxic to cancer cells, its activation potential to sensitize low responsive MDR cancer cells will have a major impact on improving the clinical performance of chemotherapy. Studies were conducted to determine synergy between MsDef1 and Doxorubicin drugs using cell death quantification. For example, MsDef1 (e.g., ~25 μM) and Doxorubicin (1mg/mL) showed ~30% cell death individually compared to untreated controls (**Figures 7A–C** or **7E–G**) in both MDA-MB-231R and MCF-7R cancer cells. However, when the cancer cells were pretreated with 25 μM MsDef1 followed by treatment with 1mg/mL Doxorubicin, a synergistic increase in cell death (>75%) was observed as compared to that observed for Doxorubicin or MsDef1 treatment alone (**Figures 7D–H**). This is true for both MDA-MB-231R triple negative breast cancer cells and MCF-7R breast cancer cells. In viability assays, a combination of MsDef1 and Doxorubicin had IC_50_ value ~10-fold lower than that of Doxorubicin (**Figure 6C**). Synergistic cell death was also verified by calculating the combination index (CI) values using Chaou-Talalay method^41.^ (**Figure 6D**). Combination index value suggested synergy (CI <1.00) and not just additive activity (CI =1). CI values were obtained over a range of fractional cell kill levels (*i.e.*, 0.05 to 0.95; 5-95% cell kill), and demonstrated values <1 for two dose combinations of MsDef1 and Doxorubicin (**Figure 6D**, blue and red lines). This is the fourth premise on which MsDef1 is proposed as a targeted tumor sensitizer.

### MsDef1 stability to protease digestion

**Figure 8:**
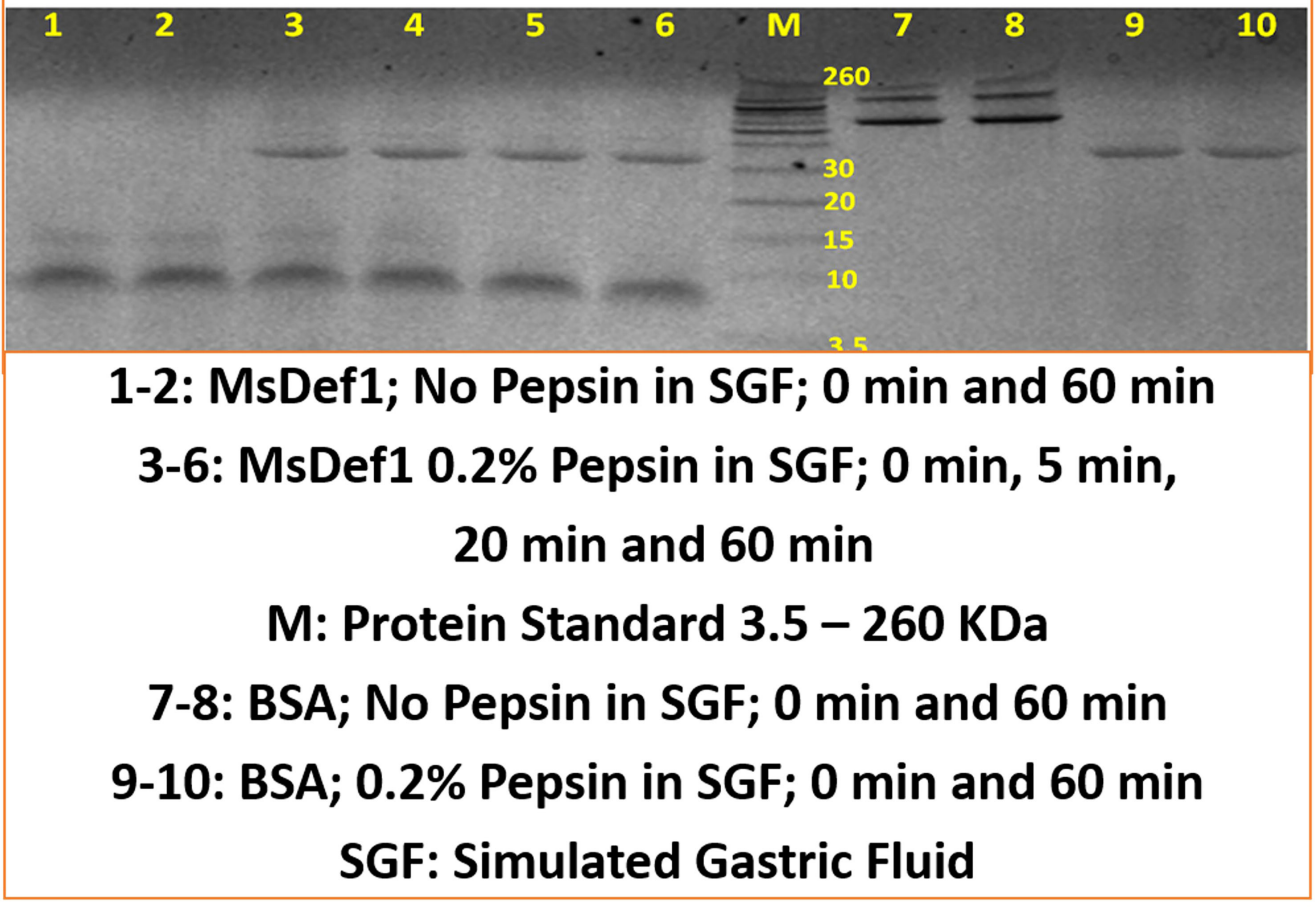
MsDef1 stability in Simulated Gastric Fluid (SGF, *in vivo* mimicking conditions) while, positive control BSA degraded, n=4, p <0.08.

The most important parameter that determines drug efficacy and safety is the stability of drug “*in vivo*”. The stability of MsDef1 to digestion by proteases was assessed by incubating MsDef1 with pepsin simulated gastric fluid (SGF) to predict it’s *in vivo* stability. MsDef1, in presence of SGF, remained undigested (lanes 3-6 compared to lanes 1-2 in **Figure 8**), while positive control BSA (lanes 9-10 in **Figure 8**) was completely degraded. This result suggests high stability of MsDef1 towards protease digestion *in vivo*.

## Discussion

The antifungal properties of plant defensins, particularly MsDef1 are well studied by our collaborator Shah et al. (36–38) in plant science. However, their potential as anti-cancer agents remain largely underexplored. We have previously reported that the antifungal mechanism of action (MOA) of the plant defensin MsDef1 involves its potential interaction with GlcCer (38). In order to characterize the role of this interaction with cancer cells, we first determined the three-dimensional structure of MsDef1 using nuclear magnetic resonance (NMR) similar to the homology-based three-dimensional structure of MsDef1 reported earlier (51) and NMR structures of several other plant defensins (51). The structure of MsDef1 consists of one α-helix and a β-sheet consisting of three anti-parallel strands and adopts the cysteine-stabilized α/β fold. In this study, we used NMR to analyze the conformation and dynamics of MsDef1 in presence of DPC micelles and DPC micelles plus GlcCer extracted from the cell walls of *F. graminearum*. The ^15^N longitudinal relaxation (T1), ^15^N-^1^H NOE and chemical shift identified amino acid residues 12-20 and 33-40 as the binding sites for this sphingolipid of the uniformly ^15^N-labeled peptide. These two binding sites are located in the Loop1 and Loop2 regions of the MsDef1 structure, respectively. Similar interaction of the plant defensin Psd1 with GlcCer has been reported previously (66, 67). Binding of MsDef1 with GlcCer is the first step in mediating cytotoxicity in cancer cells and is the first premise on which we rationalized the utility of MsDef1 as a potential tumor sensitizer for cancer therapy. The interaction of MsDef1 with GlcCer enabled us to predict and propose the potential utility of this peptide in cancer therapeutics.

The MDR cancer cells is a major problem in the treatment of cancer. For example, Doxorubicin which is a front-line treatment for TNBC patients mediates cell death through the ceramide pathway. However, cancer cells circumvent the benefits of the therapy by GlcCer using the GCS enzyme converting lethal ceramide to innocuous GlcCer (25). GlcCer is established as a biomarker for MDR (23). Hence, targeting GlcCer may be a novel and innovative strategy for addressing clinical MDR issues. MDR cancer cells (e.g., MDA-MB-231R) are characterized by the overexpression of GlcCer on their surface. Jacek et al. (40) successfully extracted and quantified GlcCer from Doxorubicin resistant MCF-7R tumor cells using chromatography. MsDef1 has a net positive charge of +4 but has potential to carry a net charge of up to +7 at low pH conditions. The anionic character of the plasma membranes of cancer cells brought about by externalization of phosphatidylserine could facilitate high binding of cationic MsDef1. Our NMR studies (**Figures 1**, **2**) established that specific residues of MsDef1 binding to GlcCer mediating intracellular cytotoxic effects.

Ceramide pathway is involved in mediating cytotoxicity of anthracyclines. Our results on resistant TNBC cells showed the release of ceramide from GlcCer (**Figure 3**). This is in contrast to the conversion of apoptotic ceramide to non-apoptotic GlcCer, a biomarker of MDR by cancer cells. In other words, MsDef1 treatment could revamp the ceramide pathway inhibiting one of the resistance mechanisms. Our data showed that MsDef1 induced sphingomyelinase activity and enhanced ceramide levels in resistant MCF-7R cells (**Figure 3B**). Since ceramide is a powerful cell death inducer, the ceramide levels were correlated to the apoptosis (**Figure 3A**). The importance of the restoration of ceramide pathway lies in its ability to modulate the biochemical and cellular processes that lead to apoptosis. MsDef1 was also compared with α-Tocopheryl succinate (α-TOS) which mediates its cytotoxic effect through ceramide pathway (53). A significant increase in the ceramide release by MsDef1 in resistant cancer cells suggests that this peptide might be able to create structural defects on the surface of cancer cell membranes by cleaving the bond between ceramide and glucose. A potential permeation mechanism is proposed based on confocal studies (see below).

Equally important in the development of MDR is the overexpression of Trx as a defense mechanism which occurs in response to oxidative stress during chemotherapy treatment. High Trx levels are directly correlated with inhibition of the endogenous ASK1 pathway making chemotherapy ineffective. Tumors with low Trx levels exhibit a better prognosis than tumors with high Trx levels (poorer prognosis, *P* < 0.001) for partial free survival (PFS) and for overall survival (OS) (32, 33). In fact, Trx is a well-studied target which lowers the response rate to specific docetaxel, cis-platin and Doxorubicin treatment while sensitizes cancer cells to Perifosin once Trx is inhibited (68). Oxidation of Trx releases ASK1 bonded to Trx. Our data clearly showed oxidation of Trx by 20 μM MsDef1 as compared to 2 mM H_2_O_2_ demonstrating a better oxidation potential of MsDef1 than H_2_O_2_. Oxidation of Trx is known to activate ASK1 cell death pathway and sensitizes tumor cells (68). The presumption that Def1 permeates MDR tumor cells is true because MsDef1 could access intracellular Trx. Similarly, creation of pores on the membrane surface of GlcCer positive MDR cancer cells by MsDef1 may also be rationalized since MsDef1 got the access of intracellular Trx in tumor cells, despite more research is needed to corroborate it. This result agrees with the finding that human β-defensin1 (hBD-1) similar to MsDef1oxidizes Trx *in situ* (69, 70) and unmasks its biological activity. For example, after reduction of disulphide-bridges hBD-1 becomes a potent antimicrobial peptide against opportunistic pathogens. Similarly, MsDef1 presumably becomes anti-tumorigenic after it got reduced to a potent form by tumor specific Trx. It is observed in our studies that all cancers cells were Trx positive while normal cells, cardiomyocytes and bone marrow cells were Trx negative. These data also indicate a potential strong selectivity of MsDef1. Similarly, Trx inhibitors (e.g.PX12) are known to oxidize Trx to reactivate ASK1 pathway and sensitize cancer cells to chemotherapy (68). Targeting ceramide and ASK1 dual cell death pathways is the hallmark of MsDef1 which may be unique compared to the other existing cancer treatments.

Interaction of MsDef1 with the tumor specific MDR biomarker Trx may have several implications for the potential trapping of MsDef1 inside cancer cells. This may be important for the continuous activation of ASK1 cell death pathway resulting in the antitumor properties and/or synergy of MsDef1 with the existing treatments. Defensins are cationic at low pH with cell penetrating properties [e.g., human β-defensin, hBD-1 (69, 70)]. In this study, we tested the cell penetrating ability of MsDef1 in MDR tumor cells using the NBD-conjugated peptide. Confocal microscopy studies performed using MsDef1-NBD incubated with TNBC MDA-MB-231R and ovarian SKOV3 cells showed the uptake of the peptide, although not linearly to different concentrations. It appears that there is a significant change in the intensity of fluorescence signal from 0 mg/mL to 2.22 mg/mL to 6.6 mg/mL and get saturated at higher concentrations. The low uptake of MsDef1-NBD by tumor cells established the selectivity of the peptide to tumor cells. BODIPY-labeled plant defensin NaD1 was also reported to localize in organelles of lymphoma U937 cells (66, 67) demonstrating the cancer cell penetrating ability of plant defensins.

We hypothesized that MsDef1 with its ability to penetrate GlcCer positive cancer cell membranes could potentially increase the diffusion of Doxorubicin into the tumor. This hypothesis was corroborated by measuring the influx of Doxorubicin by MsDef1 in MDR cancer cells through the intrinsic fluorescence of Doxorubicin at 488 nm measured using confocal microscopy (**Figure 5G**). The low intensity of fluorescent signal by Doxorubicin alone confirms the reported poor tumor penetrating ability of Doxorubicin. The higher influx of Doxorubicin indicated by the higher intensity of fluorescent signal by cancer cells which are pretreated with MsDef1 implies that MsDef1 may permeate MDR cancer cells making Doxorubicin diffuse better into the tumor cells.

The inhibitory activity IC_50_ of MsDef1 against several GlcCer and Trx positive cancer cells is in the 10-15 μM range which is similar for many chemotherapeutics (62–65) making MsDef1 clinically viable. The interesting part is that IC_50_ MsDef1 is 15-20-fold more in normal epithelial breast cells and more importantly in cardiomyocytes (Table in **Figure 6A**). In contrast, Doxorubicin has IC_50_ very low at 9.6 μM in cardiomyocytes which makes it cardiotoxic (**Figures 6A, B**). However, Doxorubicin combined with MsDef1 was ~25x more potent, against MDA-MB-231R cancer cells confirming the synergy between MsDef1 and Doxorubicin (**Figure 6B**). The data was further corroborated with the calculation of combination index (CI) using Towley method (61) which showed CI values less than 1.00 indicative of synergy and not just addition (6D). Although, several defensins (71) have been reported to be cytotoxic to cancer cells (Table in **Figure 6A**) they shared little sequence homology with MsDef1 and none of them were shown to bind GluCer, permeate cells, synergize with Doxorubicin, and hit the intracellular tumor specific target (Trx). This was the fourth premise on which MsDef1 is proposed as a targeted tumor sensitizer.

The risk of anthracycline related cardiomyopathy increases with a higher cumulative anthracycline dose (72, 73). About 3% of that dose persist even after the completion of therapy leading to potential heart failure and death for a dose of 400 mg/m^2^, 7% for a dose of 550 mg/m^2^, and 18% for a dose of 700 mg/m^2^. The poor tumor penetration capacity by Doxorubicin and its off target hyperactivation of endogenous PARP in heart leads to cardiomyopathy (74). Hence, improving the clinical performance of Doxorubicin may pave the way for new combination adjunctive therapy. *A priori* activation of apoptosis pathways of tumor (AAAPT) technology developed by us (39) involves targeted natural tumor sensitizers, small molecules and defensins to sensitize specifically desensitized resistant tumor cells to evoke a better response from Doxorubicin (39). In other words, sensitizing MDR cells and making them better responsive to chemotherapy is expected to make chemotherapy work at lower doses without compromising on the efficacy on tumor regression, yet reducing the cardiotoxicity to minimum by dose reduction. This synergistic approach may facilitate the expansion of the therapeutic index of the drug. Both Def1 (25 μM) and Doxorubicin (1mg/mL) induced cell death in resistant TNBC MDA-MB-231-R cells reasonably assessed through morphology of cell death (**Figure 7**). However, when combined together, the cumulative cell death was greater than just adding the two drugs. The change in IC_50_ for the combination is ~25 times higher compared to individual drugs (**Figure 6C**, from 394.6 nM to 16.5 nM for the combination). This translates, clinically that a combined formulation potentially may reduce the tumor burden at a lower Doxorubicin dose which in turn may reduce the dose related toxicity induced by Doxorubicin, particularly cardiotoxicity. It is to be noted that MsDef1 does not tackle the Doxorubicin toxicity directly. We infer that cardiotoxicity of Doxorubicin is tackled by making it work effectively at lower doses which automatically lowers dose related cardiotoxicity. In other words, physicians may have a larger window for fine tuning the combination dose regimen based on the potential combined lower toxicity rather than the individual toxicities. MsDef1, being natural and expressed in corn and alfalfa is presumably not expected to be toxic at the clinical dose levels, although not proved *in vivo* so far. However, FDA considers MsDef1 belongs to the GRAS (generally regarded as safe) category. It is possible to leverage this by changing MsDef1 dose in a larger window and reducing the Doxorubicin dose for potential, combinatory synergistic effects. In our recently published work (40) on small molecules we have established in a rat tumor model that the ejection fraction measured through US imaging was retained for the combination (> 65%) while it did not for Doxorubicin alone (< 45%).

Two major requirements for the translation of bench concept to clinical product are a) stability of MsDef1 and b) selectivity of MsDef1 to cancer cells. In general, MsDef1 type of molecules are quite stable in media due to the cyclization of cysteines. Stability of MsDef1 *in vivo* towards proteolytic enzymes may not be a big concern since several tetra sulfide array compounds including FDA approved products developed by Pandurangi et al. (75, 76); (e.g., NeoTect, NeoTide), human beta defensin (hBD-1) with three S-S bonds (69, 70), cyclized cyclotides (77), θ defensins (78) provide solid examples of high stable compounds similar to MsDef1. Typical concentration of defensin involved in host defense against microbial infections range ~ 10mg/mL (e.g., granules, intestine) indicates reasonable stability *in vivo* (79). Preliminary studies on the stability involved incubation of MsDef1 in simulated gastric fluid (SGF) which mimics *in vivo* conditions, and the stability was assessed through Western Blotting. MsDef1 showed no significant change in the intensity of the bands with respect to time, while positive control BSA was degraded easily (**Figure 8**). This implies high stability of MsDef1 towards protease digestion. The combination of a cyclic cysteine knots (CCK) and a circular backbone renders peptide impervious to enzymatic breakdown exemplified by cyclotides (77), theta defensins (78) and FDA approved drugs like AcuTect (75, 76) and NeoTect (75, 76).

**Scheme 1:**
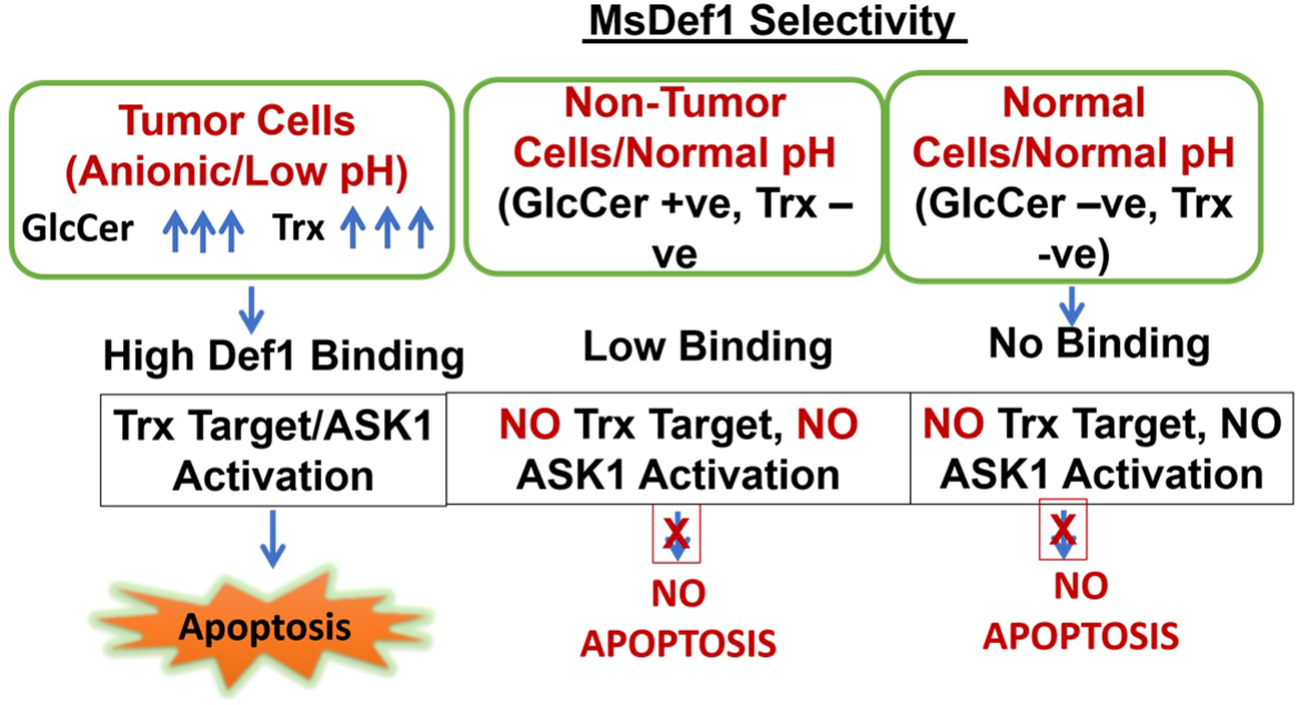
Selectivity of MsDef1.

**Scheme 2:**
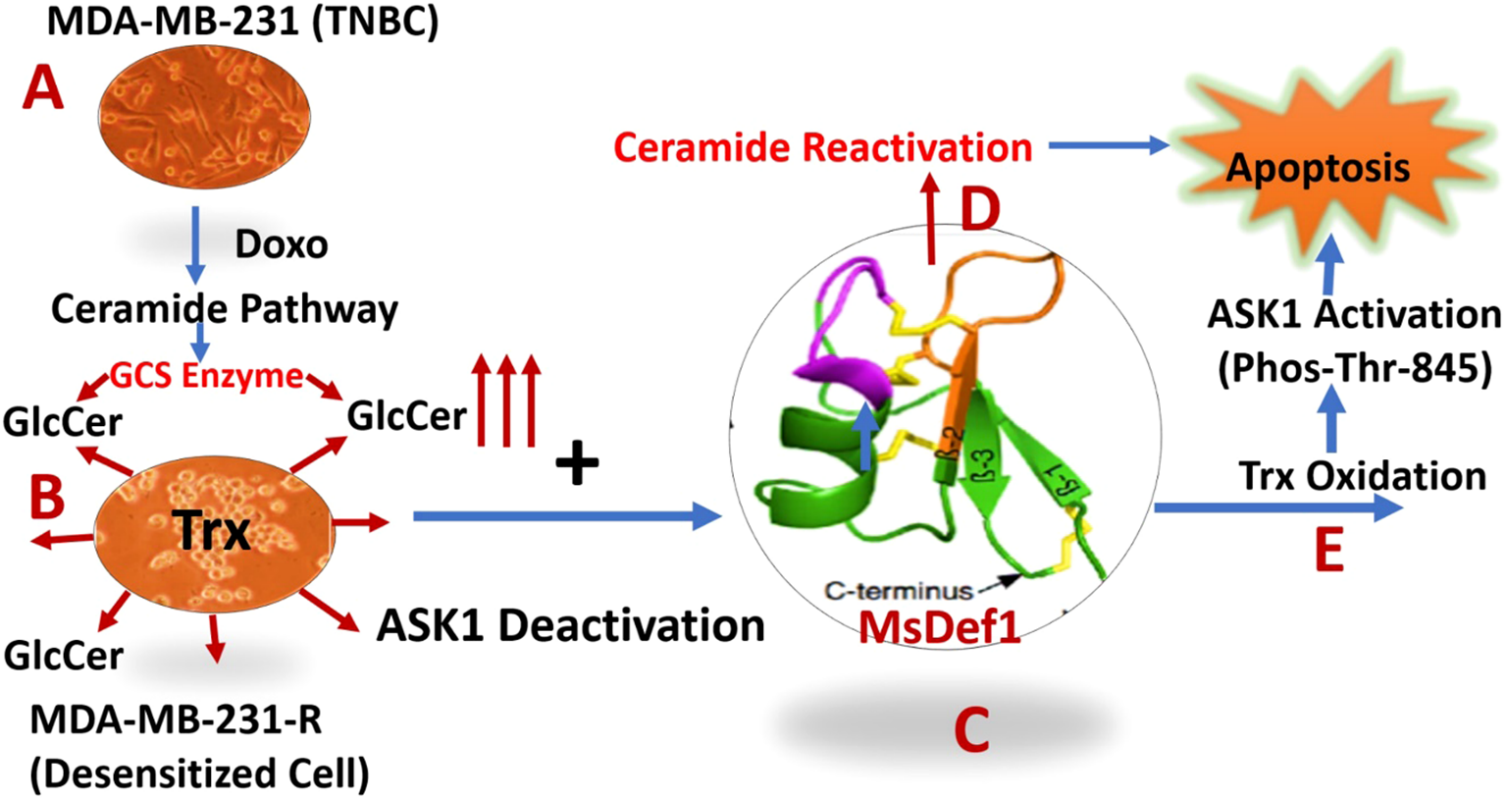
Description of dual Pathways activation by MsDef1 which sensitizes multidrug resistant (MDR) cancer cells: **(A)** Doxorubicin activates ceramide pathway in triple negative breast cancer cells (MDA-MB-231), while cancer cells circumvent it by overexpressing Glucosilceramide Synthase (GCS) which glycosylates ceramide to convert it to Glucosilceramide (GlcCer) which is MDR biomarker, **(B)** Deactivated MDR-MB-231R MDR cancer cells overexpress Thioredoxin (Trx) deactivating Apoptosis Stimulating Kinase 1 (ASK-1) pathway, **(C)** MsDef1, Upon treatment liberates ceramide by reactivating ceramide pathway, **(D)** leading to apoptosis, **(E)** MsDef1 also oxidizes Trx activating ASK1 Pathway sensitizing MDR cancer cells to Doxorubicin leading to synergistic apoptosis.

MsDef1 is shown to target two tumor specific targets in this study, i.e., GlcCer and Trx. Overexpression of both GlcCer and Trx made cancer cells resistant to treatments and are considered as biomarkers of resistance. The selectivity of MsDef1 may be hypothesized as described in **Scheme 1**. Cells are categorized into 3 classes for understanding selectivity: Class A: Tumor cells which are anionic, low pH, GlcCer positive and Trx positive, Class B: Nontumor cells, GlucCer Positive, normal pH, and Class C: Normal cells, Normal pH, Both GlcCer and Trx negative. In Class A, tumor cells, particularly MDR cells have both GlcCer and Trx positivity and are also at low pH conditions. Under these conditions, MsDef1 is cationic with +7 charge which interacts with anionic sphingolipids strongly to liberate ceramide from GlcCer. Hence, it is reasonable to assume that MsDef1 might have created structural pore defects on the membrane of cancer cells allowing the diffuse of MsDef1 which in turn interacts with intracellular Trx through Trx. That liberates ASK1 protein from Trx activating ASK1 pathway. In Class B case, off target cells have low GlcCer positivity and low binding of MsDef1 to GlcCer positive cells is expected to trigger the release of ceramide. However, low levels of ceramide are expected to have little side effects. In Class C, it is obvious that these cells are both GlcCer and Trx negative leading to no uptake of MsDef1 (**Figures 5C–E**). Although, the undesired off-target expression of GlcCer and Trx (e.g., brain, kidney etc.) may be of a little concern, it is to be noted that the overexpression of GlcCer and Trx in tumor compared to other cells is the key. It is the relative target Vs. non-target expression of the biomarker which makes it better selective compared to nonspecific chemotherapy. For example, high target/non-target ratio for many FDA approved targeted imaging and therapeutic drugs (e.g., Octreoscan (80), Herceptin (81)) based on somatostatin and ErbB2 overexpression respectively, are good examples of selectivity despite low expression of the biomarker at non-target sites. It is the combination of two tumor specific targets (GlcCer & Trx) and reactivation of two pathways (ceramide and ASK) which makes MsDef1 unique (**Scheme 2**).

In summary, TNBC patients treated with Doxorubicin are not getting benefitted much since ceramide pathway through which Doxorubicin mediates cell death was deactivated by cancer cells (**Scheme 2A**). The deactivation of ceramide pathway is due to the classic glycosylation of ceramide which produces MDR cancer cells. We have clearly shown that MsDef1 reactivated ceramide pathway, liberating ceramide from GlcCer, oxidizing intracellular Trx and in turn activating ASK1 pathway. As a result, MDR cancer cells are sensitized to Doxorubicin through synergy. Our studies revealed that MsDef1 could be a natural tumor sensitizer which can be useful at neoadjuvant settings with chemotherapy. Further studies are needed to test the synergicity of MsDef1 in tumor animal models *in vivo* for a potential smooth clinical translation.

## Data availability statement

The datasets presented in this study can be found in online repositories. The names of the repository/repositories and accession number(s) can be found in the article/**Supplementary Material**.

## Author contributions

RP: conceptualization, interpretation of data, writing original draft, design of experiments. DS: expertise review on MsDef1, manuscript editing. KH-W: NMR data collection, interpretation. US and AK: data collection. All authors contributed to the article and approved the submitted version.

## Supplementary material

Supplementary Figure S1 MsDef1 oxidizes Trx at 20 mM dose compared to positive control H_2_O_2_ (2 mM) in MDA-MB-231-R TNBC cells, 1. Def1 (0 mM), 2. Def1, (10 mM), 3. (20 mM), 4. H_2_O_2_ (10 mM) (N=4, p < 0.05).

Supplementary Figure S2
**(A)** SDS-PAGE of MsDef1 prepared using E-coli Rosetta (DE3/pET 28a). **(B)** 1-6 HPLC Fractions, Fraction 1 corresponds to molecular weight of fully folded MsDef1 (5183) which showed antifungal activity.

Supplementary Figure S3 Immunoblot analysis of phosphorylation of Threonine-845 residue of ASK1 Protein in response to the treatment of Def1 in MDA-MB-231 cells: 1. Control, 2. N-Acetyl-Cystein (NAC, 5 mM)/60 mins, 3. NAC (5 mM) + Def1 (50 μM ) for 60 min, 4. Def-1 (50 μM) alone for 60 minutes, N=4, P < 0.005, GAPDH: Internal Control.
