# Supplementary figures and images for: Medicago Sativa Defensin1 as a tumor sensitizer for improving chemotherapy: translation from anti-fungal agent to a potential anti-cancer agent
Source: Front Oncol. 2023 May 26;13:1141755. doi: 10.3389/fonc.2023.1141755 (PMC10251204; doi:10.3389/fonc.2023.1141755)

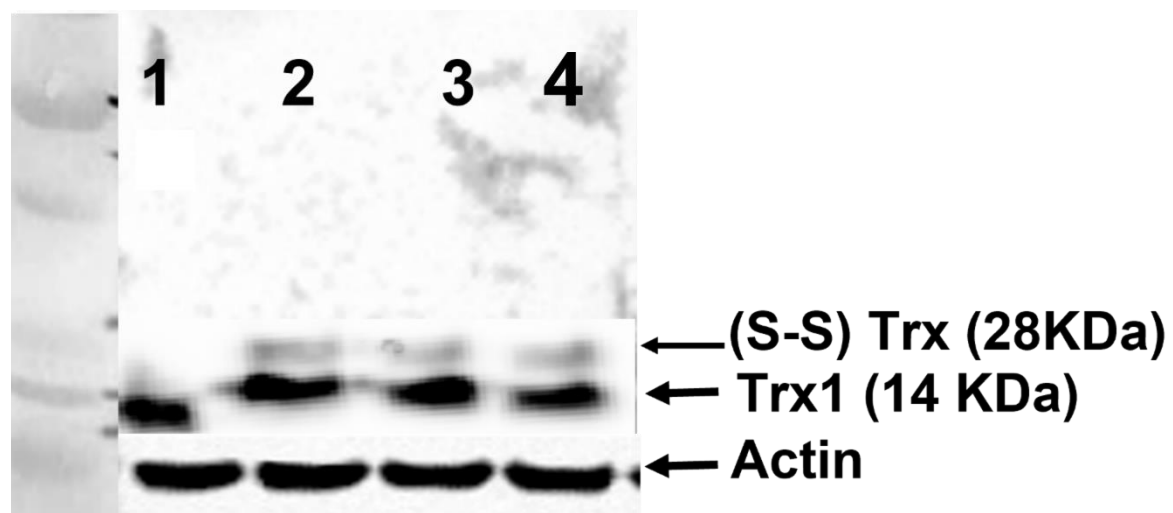

Supplement: Supplementary Figure S1 — MsDef1 oxidizes Trx at 20 mM dose compared to positive control H2O2 (2 mM) in MDA-MB-231-R TNBC cells, 1. Def1 (0 mM), 2. Def1, (10 mM), 3. (20 mM), 4. H2O2 (10 mM) (N=4, p < 0.05). [file DataSheet_1.pdf]

## Supplementary Materials

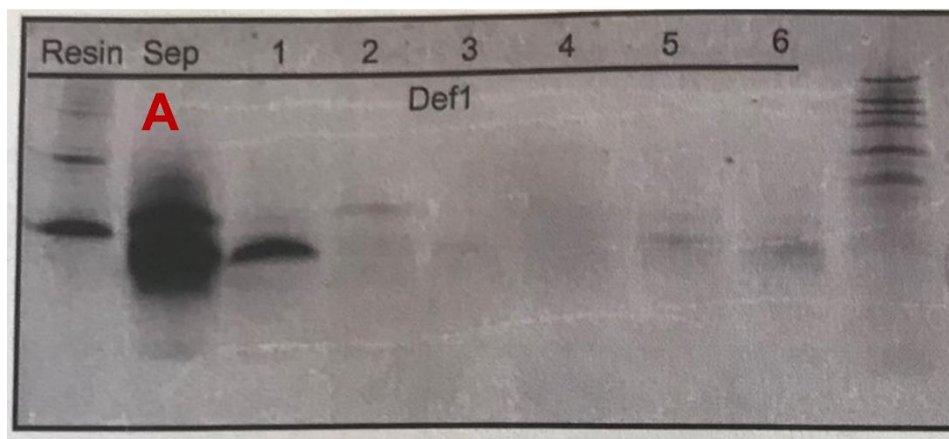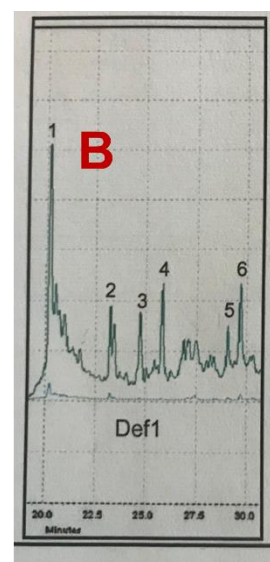

Supplement: Supplementary Figure S2 — (A) SDS-PAGE of MsDef1 prepared using E-coli Rosetta (DE3/pET 28a). (B) 1-6 HPLC Fractions, Fraction 1 corresponds to molecular weight of fully folded MsDef1 (5183) which showed antifungal activity. [file DataSheet_2.pdf]

1 2 3 4

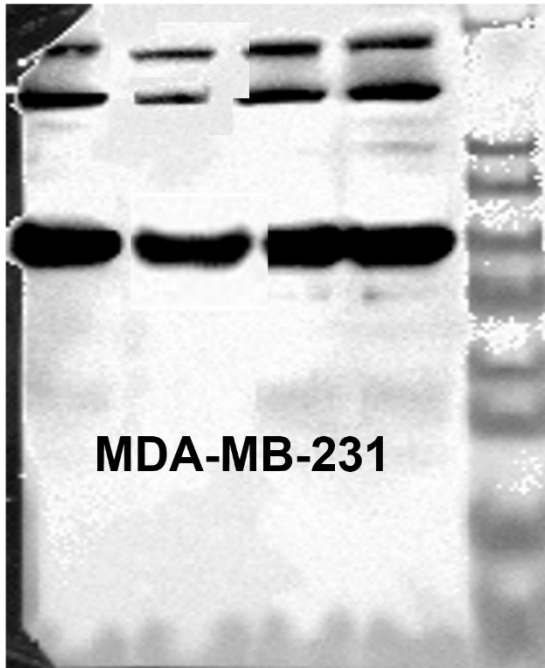

**P-Thr 845/ASK1**

**GAPDH**

**MDA-MB-231**

Supplement: Supplementary Figure S3 — Immunoblot analysis of phosphorylation of Threonine-845 residue of ASK1 Protein in response to the treatment of Def1 in MDA-MB-231 cells: 1. Control, 2. N-Acetyl-Cystein (NAC, 5 mM)/60 mins, 3. NAC (5 mM) + Def1 (50 μM ) for 60 min, 4. Def-1 (50 μM) alone for 60 minutes, N=4, P < 0.005, GAPDH: Internal Control. [file DataSheet_3.pdf]
